# Connecting the dots: integrating citizen science, ecology, and habitat change for the conservation of three Leporidae species in the central mountains of Mexico

**DOI:** 10.1007/s00114-025-02048-1

**Published:** 2025-12-01

**Authors:** Eric O Ramírez-Bravo, Evangelina E. Camargo-Rivera, C. Sánchez-Lewy Aldana, F. Amador-Cruz, M. A. Mora-Ramirez

**Affiliations:** 1 Agroecosystems Center, Sciences Institute, Benemérita Universidad Autónoma de Puebla, Puebla, Mexico; 2 Motocle A.C. San Andrés Cholula, Puebla, Mexico; 3 Dirección General de Zoológicos de la Ciudad de Mexico, Secretaría del Medio Ambiente de la Ciudad de México (SEDEMA), Mexico, Mexico; 4 Departamento de Ecología, Facultad de Estudios Superiores Iztacala, Universidad Nacional Autónoma de México, Hab Los Reyes Ixtacala, Av. de los Barrios 1, Tlalnepantla, 54090 Mexico; 5 Faculty of Chemistry, Benemérita Universidad Autónoma de Puebla, Puebla, Mexico

**Keywords:** Alpine grasslands, Izta-Popo national park, Mexico city, Volcano rabbit, Precipitation index, NDVI

## Abstract

Anthropogenic activities have a profound impact on ecosystems, threatening species and contributing to increased extinction rates. Among them are some Leporidae species, for which ecological data remains insufficient to support effective conservation. This study aimed to monitor the status of the volcano rabbit (*Romerolagus diazi*), eastern cottontail (*Sylvilagus floridanus*), and Mexican cottontail (*Sylvilagus cunicularius*) in 2018 in southern Mexico City, using citizen science tools. The field research involved recording fecal pellets and habitat characteristics along 1 km transects. Vegetation changes over the past 20 years were analyzed using the Standardized Precipitation Index (SPI), Enhanced Vegetation Index (EVI), and Standard Anomaly Index (SAI). Among 2,026 quadrants (~ 8,000 km²), the volcano rabbit, eastern cottontail, and Mexican cottontail were detected in 3.25%, 8.09%, and 12.37% of sampling points, respectively. Canonical correspondence analysis revealed that mature tree density harmed the presence of the volcano rabbit. At the same time, cottontails appeared to be more resilient to habitat changes. Despite reforestation efforts, SPI, EVI, and SAI data indicated an increase in dryness over time. Intensive planting has transformed alpine grasslands, a key habitat for the volcano rabbit, into densely forested areas. Citizen science is an effective tool for monitoring certain species of leporids, such as the volcano rabbit, and provides valuable insights for conservation strategies. Future interventions should prioritize the preservation of alpine grasslands to ensure their long-term survival.

**Supplementary Information:**

The online version contains supplementary material available at 10.1007/s00114-025-02048-1.

## 1. Introduction

Human activities impact ecosystems, threatening natural areas that protect specific species (Sanderson et al. 2002) and increasing extinction rates (Pimm et al. 1995). Conservation science aims to mitigate the factors that impact species and ecosystems (Redford et al. 2011) by implementing proposed conservation policies, plans, and actions (Lughadha et al. 2020). However, to define the more plausible conservation tools, it is necessary to correctly evaluate the additional risks and factors that threaten each species (Venter et al. 2006), especially for small-range distribution species, which have been suggested as the most suitable to identify priority areas for conservation (Veach et al. 2017). The taxonomic family of Leporidae species has been an excellent study object with positive conservation actions (Smith 2008). However, protecting these highly isolated species is quite a complex challenge. In this regard, the volcano rabbit (*Romerolagus diazi*), also known as “Teporingo” in Mexico, is a species categorized by the International Union for Conservation of Nature and Natural Resources as endangered (IUCN 2015). Volcano rabbit is an endemic Mexican species distributed in the Trans-Mexican Neovolcanic Belt and currently inhabits only four volcanoes (Popocatepetl, Iztaccihuatl, Pelado, and Tlaloc), where it coexists with other large-distribution lagomorph species, like Mexican cottontail (*Sylvilagus cunicularius*) or “Montes” as known in America Latina and eastern cottontail (*S. floridanus*) called “Castellano” in Mexico. These species of Leporidae face several human threats, including habitat destruction for agricultural purposes and road construction that divides core populations (Smith et al. 2018), resulting in reduced connectivity and, consequently, limited genetic flow (Velazquez 1993; Hunter and Cresswell 2015). The total distribution of the volcano rabbit is limited to ≈ 386 km^2^ (Smith et al. 2018) due to its specialized habitat preferences, including open pine forests (*Pinus hartwegii*) and dense bunchgrass communities (*Festuca tolucensis*) (Hoth and Granados 1987; Cervantes et al. 1990; Hunter and Cresswell 2015). It is noteworthy that in Mexico, the volcano rabbit started receiving attention from different institutions in the 80 s (Bell et al. 1985), generating information about its distribution (Rizo-Aguilar et al. 2015), habitat preferences (Uriostegui-Velarde et al. 2018), and feeding habits (Hoth and Granados 1987). However, this knowledge production has been useless in stopping the decline of their populations, estimated at 11,000 to 25,000 individuals (Velazquez and Gopar-Merino 2018). Besides, being hunted by local communities, the interaction of the volcano rabbit, Mexican cottontail, and eastern cottontail with other species is usually unfavorable for the leporids; for instance, they are hunted by feral cats and dogs (Hunter and Cresswell 2015), that most of the time also carry diseases that pose a significant threat to the species (Velazquez and Heil 1996). Likewise, climate change is another potential threat to the species due to changes in vegetation in high-elevation habitats (Jiménez-García et al. 2021). For the above, reducing the knowledge gap associated with interacting these three Leporidae species with habitat variables and how climate and vegetation changes could affect their populations is fundamental. Implementing citizen science helps fill these information gaps (Strasser et al. 2019) as it develops behavior and attitudes, “the identity of appropriation” in the participants (Kelemen-Finan et al. 2018), to promote the generation of a complete conservation plan. Whereby this study adds to the knowledge of leporids: (*i*) the effectiveness of incorporating people to monitor endangered species, (*ii*) evaluates the habitat characteristics involved in the distribution of the three sympatric leporid species (volcano rabbit, Mexican cottontail, and eastern cottontail) present in the area at a small-scale level and (*iii*) analyze rainfall and vegetation variations over the last 20 years to determine the possible threat for the leporids.

## 2. Methodology

### 2.1 Study area

The study was conducted on the Pelado Volcano in Mexico City (19.20º N, −99.14º W) during the dry season of October-November 2018, covering approximately 8104 km^2^. This area is part of the Ajusco National Park, situated in the southern region of Mexico City, bordering the State of Morelos. To the north, Pelado Volcano shares boundaries with Ajusco, San Mateo Xaloa, and Santiago Tepecatlapan; to the south, with Parres and Huitzilac; to the east, with Sierra de Chichinahutzin and San Francisco Tlalnepantla; and to the west with Magdalena Petlacalco. The habitat characteristics in this region promote the leporid species, particularly the volcano rabbit (Rizo-Aguilar et al. 2015). The elevation in the study area ranges from 3000 to 3500 m.a.s.l., with a mild to cold climate, distinct rainy and dry seasons, an average temperature of 11 °C, and a mean annual rainfall of 1000 mm (Velazquez 1993). Approximately 90% of the study area is comprised of fir, oak, and pine forests. Grasslands, known as “zacatonales”, occupy 5.5% of the area, and are characterized primarily by species of *Muhlenbergia* spp., *Festuca* spp., and *Stipa ichu* (Rizo-Aguilar et al. 2015). Also, in minor proportions are zones of farmland (2.8%) and temperate forest (1.2%). Figure A1 shows the spatial distribution of vegetation and areas used for farmland, mainly devoted to oat and potato cultivation, which represents a threat to the habitat of the Leporidae species.

## 2.2 Community monitoring

Citizen science projects offer an opportunity to enhance citizens’ knowledge and foster positive attitudes toward science, particularly those with opportunistic data collection, minimal or no sampling design, and limited volunteer training (Brown and Williams 2019; Bruckermann et al. 2021). Additionally, locals can recognize, name, and interpret ecological interactions among locally occurring species, regardless of whether these species benefit them economically (Nabhan 2000). In the area of study, there have been citizen science projects that provide data about the distribution and conservation status of species, for instance, the project of Sierra Madre Sparrow (*Xenospiza baileyi*) by Ortega-álvarez et al. (2020). Thus, including people from local communities can boost conservation efforts in the area. In this paper, a monitoring brigade composed of twenty people from the community members from San Miguel Topilejo conducted the assessment from September to November 2018. This group was heterogeneous in terms of age and educational level, ranging from primary to high school. In terms of experience, only one of the monitors had experience in monitoring Leporidae species. However, for most, this project was their first opportunity to collaborate. There were two stages preceding the field campaign. In the first stage, two workshops were held to provide brigade members with training on monitoring the volcano rabbit, Mexican cottontail, and eastern cottontail species, as well as undertaking quadrats to monitor vegetation. The workshops also defined biodiversity, its importance, monitoring methodologies, data analysis, and possible applications. The second stage involved practical teaching, including monitoring methods used during the project, techniques for data registration, and, of course, the identification characteristics of the species. Both stages were conducted near the communities; games and ludic activities were included at these meetings to facilitate learning, promote long-term retention, and encourage attitude change.

## 2.3 Sampling method

Leporids were challenging to see due to their ecological habits (Cervantes et al. 1990), so the presence of species was determined using fecal pellet identification; these were distinguished among different species (*R. diazi*,* S. floridanus*, and *S. cunicularius*) using pellet color and shape (Aranda Sanchez 2012). During the period of study in 2018, a total of 21 transects were randomly selected across the study area, each 1 km in long. For each 20 m, a quadrant of 4 m^2^ was delimited, where coordinates, elevation, and presence or absence of fecal pellets were recorded (Osuna et al. 2022). The habitat variables, such as grassland percentage, number of young and adult trees, percentage of bare soil, pine leaves in soil, and rock, are considered factors affecting the relative abundance of leporids (Osuna et al. 2022). Therefore, a 10 m radius circle was established by transect to identify possible environmental pressure factors to the species, like signs or traces of human activity, including reforestation, deforestation, firewall breaches, open-air dumps, material extraction, houses, roads, agriculture, and fires, were also recorded as presence or absence data (Lorenzo and Lanier 2019).

### 2.4 Analysis of the presence of species and habitat

The points with fecal pellet counts were used to confirm the presence of the corresponding species. The distribution of each species was delimited by round buffers (circles). The presence of species points was located using a GPS and later digitized on a map of the study area. As the volcano rabbit is a highly specialized endemic species with a marked preferred condition (Velazquez and Gopar-Merino 2018) defining the habitat variables that limit its presence is relevant. In this sense, the behavior of the leporid species with the aforementioned variables was defined through simple line graphics. Furthermore, using multivariate canonical-correlation analysis (CCA), the relationships between the three species and all habitat variables: elevation, grassland percentage, number of young and adult trees, human activity, and percentage of bare soil, pine leaves in soil or rock, were determined (McGarigal et al. 2000). The CCA excluded the position and transect variables from the model to remove autocorrelation and simplify analysis for biological reasons.

## 2.5 Relevant climate variables trends

One of the primary problems in peri-urban areas is habitat loss, which affects species distribution and densities (Mason 2000; Rebolo-Ifrán et al. 2017; Luna et al. 2018). Moreover, climate change negatively impacts water availability, leading to increased dry periods with higher temperatures (Tesfaye et al. 2019). However, there is no data on how it could affect the leporids population. One approach to determining droughts is the use of precipitation indicators, such as the Standardized Precipitation Index (SPI) (Smakhtin and Hughes 2007). Besides, it is possible to evaluate the effects of droughts on vegetation patterns using the Normalized Difference Vegetation Index(NDVI Wang et al. 2010) and the Enhanced Vegetation Index (EVI), which is similar to NDVI but optimized, since it includes corrections to reduce the influence of the atmosphere and the ground (Huete et al. 2002). The SPI measures the deviation from the distribution function of rain probability. Hence, SPI has gained importance recently as a potential drought indicator that permits comparisons across space and time (Mckee et al. 1993). A 20-year daily time series of precipitation accumulations over the desired time scale and region was used to estimate an appropriate probability density function to calculate the SPI. The analyses were based on the Pearson Type III distribution (i.e., 3-parameter gamma) as suggested by Guttman (1999). The cumulative probability distribution was then estimated and transformed into a normal distribution. The result was the SPI, which can be interpreted as a probability using the standard normal distribution (i.e., users can expect the SPI to be within one standard deviation of the mean about 68% of the time, two standard deviations about 95% of the time). The analyses utilized the *SPEI package* available in R (v2023.12.1) as used in Vicente-Serrano et al. (2010). The SPI was compared with changes in grassland and shrubs over the study area. In this regard, the Standard Anomaly of the NDVI (SAI) was calculated as follows (Pei et al. 2019):

Where *NDVI*_*mm*_ represents the monthly mean of the Normalized Difference Vegetation Index (NDVI) variable, *NDVI*_clim_ is the corresponding mean average from 2000 to 2020 and, *NDVI*_seas_ is the corresponding quarterly mean of NDVI: March to May (MAM), June to August (JJA), September to November (SON), and December to February (DJF). It is worth noticing that three climatic seasons have been identified in Mexico: (*i*) cold-dry season (DJF), (*ii*) hot-dry season (MAM), and (*iii*) rainy season (JJA, SON). In this regard, the climatology analysis is performed on a quarterly basis. After the SAI calculation is performed, the other parameter used to compare the changes in grassland and shrubs over the study area is the Enhanced Vegetation Index (EVI) because this parameter is a good proxy of leaf area index, biomass, and canopy cover, then is used to evaluate the seasonal, inter-annual, and twenty-year climate variation on the vegetation structure in the context of the SPI changes (Huete et al. 2002).

1 $$\:SAI=\frac{{NDVI}_{mm}-{NDVI}_{clim}}{{NDVI}_{seas}}$$

## 3. Results

### 3.1 Community monitoring

Community monitors helped during the fieldwork and developed their activities independently of the researchers, usually in groups of more than four people. Those monitors with experience in citizen monitoring projects performed better during the activities. However, most of the participants achieved correct recording despite the multiple variables. In total, 35 monitors participated during the dry season of October-November 2018. It’s worth mentioning that no meteorological parameter measurements were taken during the monitoring campaign. However, for future field experiments, it is advisable to measure these parameters at selected locations to perform a comparison with satellite data.

## 3.2 Species distribution

**Fig. 1:**
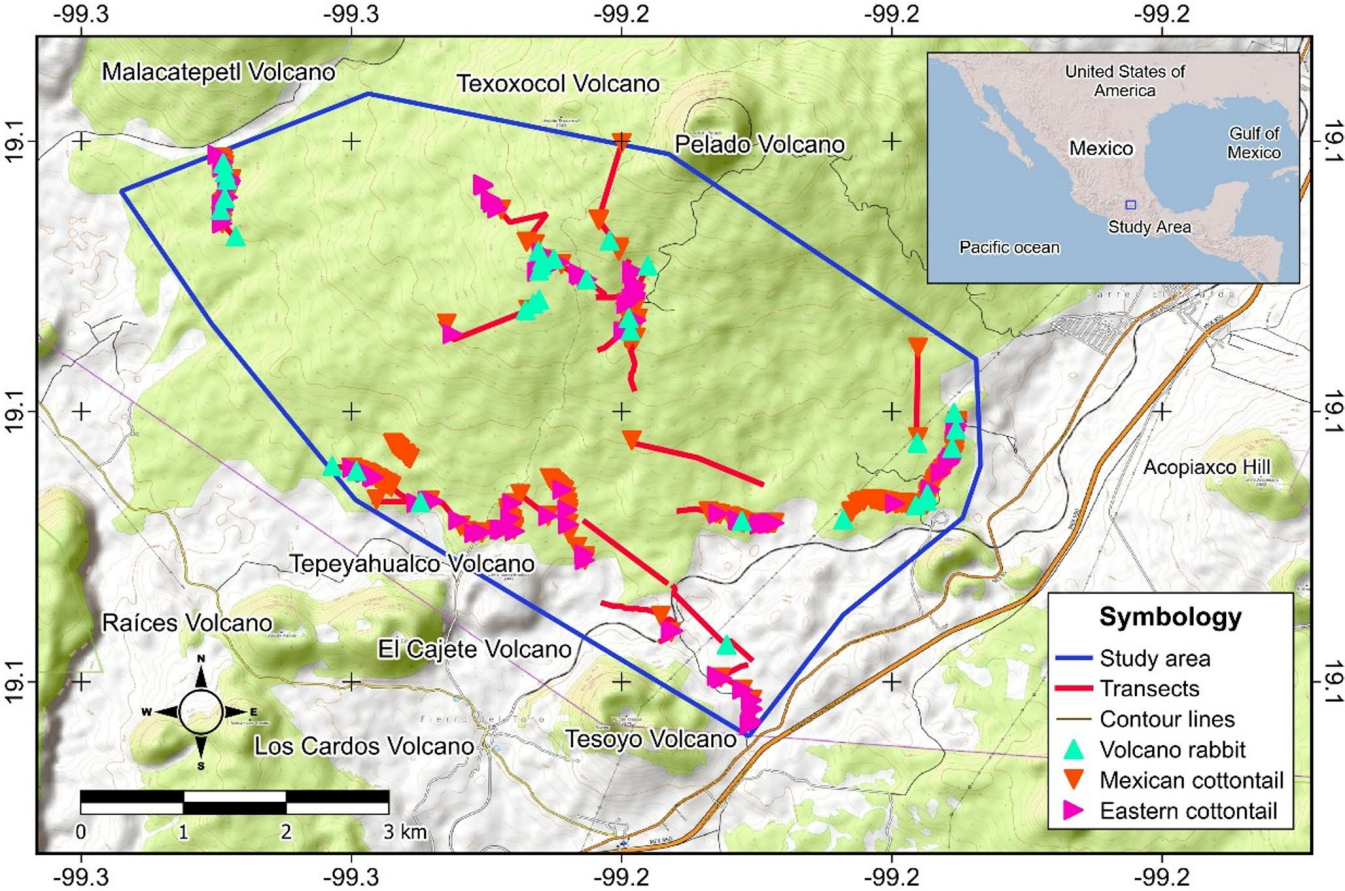
The study area (blue line) is located on the edge of Mexico City, at the foot of the mountain range (Pelado and Texoxocol Volcanoes). The spatial distribution of the volcano rabbit, eastern cottontail, and Mexican cottontail is shown

In total, 2026 quadrants of 4 m^2^ were monitored in the twenty-one transects. The volcano rabbit was the scarcest; its fecal pellets were found in only 66 sampling points (3.25%). Eastern cottontail presence was found in 164 sampling points (8.09%), and the Mexican cottontail rabbit was recorded at 271 sampling points (13.37%). The spatial distribution varied by species; the overlap between the three species’ distributions was 0.49%, while the overlap between the Mexican and eastern cottontail distributions was 2.71%. The spatial distribution of the volcano rabbit, Mexican cottontail, and eastern cottontail is shown in Fig. 1.

**Fig. 2:**
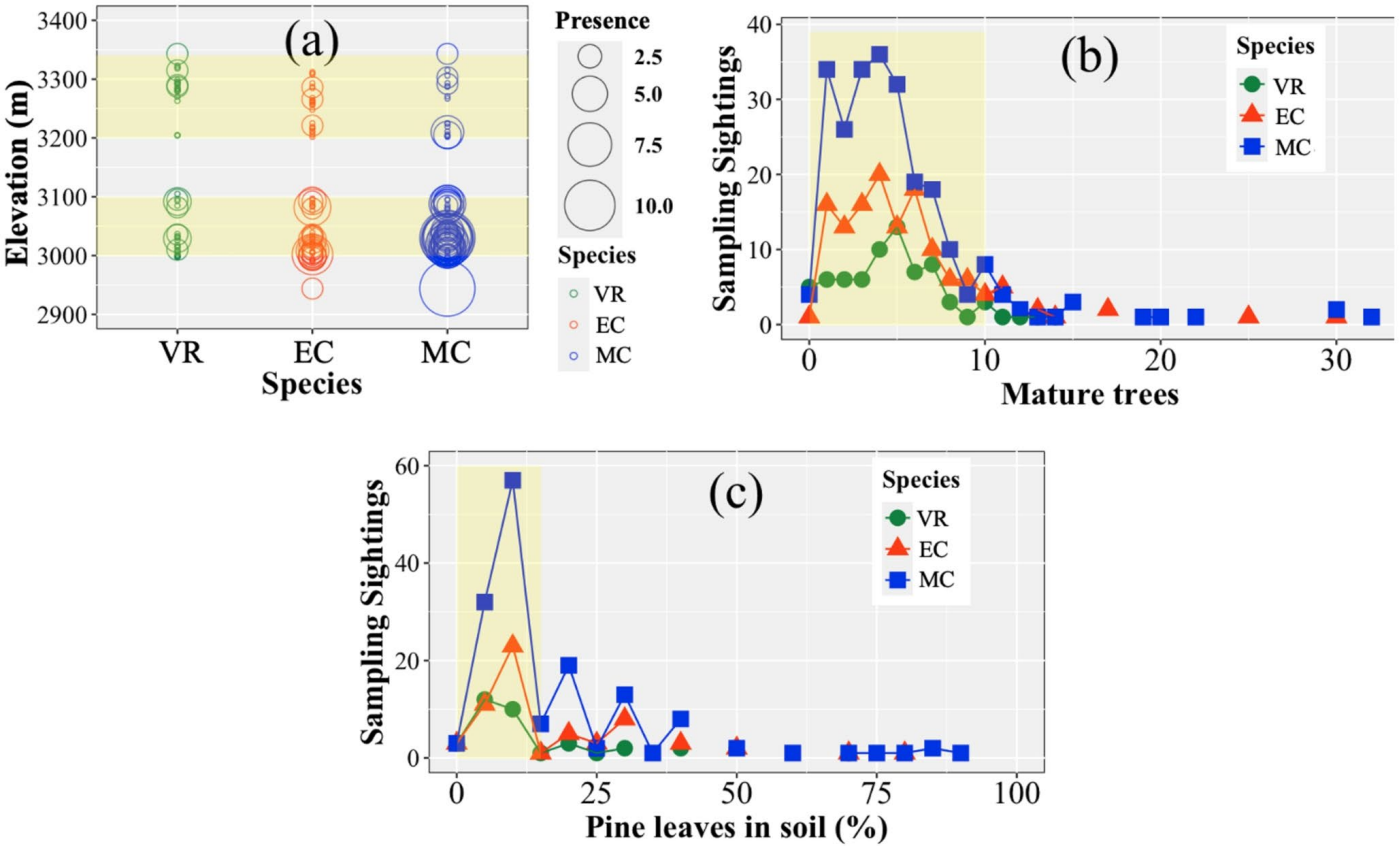
Signs of volcano rabbit (VR), eastern cottontail (EC), and Mexican cottontail (MC) as a function of the (**a**) elevation, (**b**) number of mature trees, and (**c**) percentage of pine leaves in soil inside a 10 m radius circle by transect. In Figure (a), the size of the circles corresponds to the number of fecal pellet samples found; the smaller size corresponds to 2.5, and the bigger one to more than 10 fecal pellet samples found

Furthermore, a significant difference was found between habitat variables for the three species. Figure 2 summarizes the signs or collected fecal pellets of the volcano rabbit, eastern cottontail, and Mexican cottontail as a function of the habitat variables: (a) elevation, (b) number of mature trees, and (c) percentage of pine leaves. As shown in Fig. 2(a), the three species were grouped into two preferential elevation intervals, identified by yellow-shaded areas, one at 3000–3100 m.a.s.l. and the other at 3200–3350 m.a.s.l. The number of signs as a function of mature trees is shown in Fig. 2(b). The preferential zones of the three species are related to the presence of fewer than 10 mature trees, represented by the yellow-shaded region; for a greater number of mature trees, the number of sightings decreases relatively fast. In other words, too many trees (> 10) reduce the probability of finding signs; this result agrees with the percentage of pine leaves in the soil, as shown in Fig. 2(c).

**Fig. 3:**
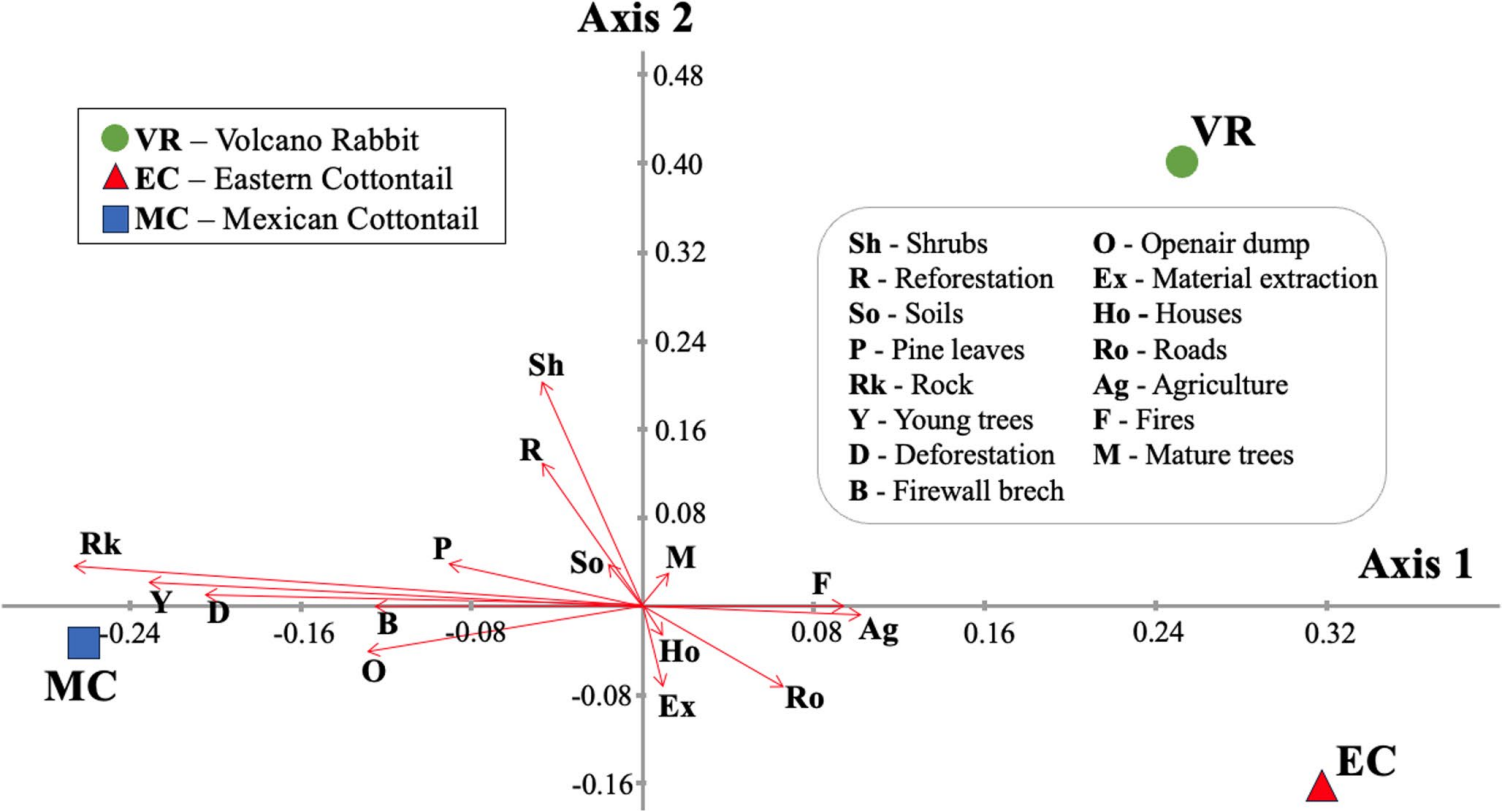
Correlation of species presence: volcano rabbit (*Romerolagus diazi;* VR), eastern cottontail (*Sylvilagus cunicularius;* EC), and Mexican cottontail (*Sylvilagus floridanus*; MC), and habitat variables explained by the first two axes of multivariate canonical-correlation analysis (CCA). The size of the red arrows indicates the total variance accounted for by each habitat variable. The habitat variables are shown in figure

The registered environmental pressure factors, or habitat threats, are summarized as follows: reforestation signs were found at 131 points (6.4%), logging at 128 points (6.3%), and, to a lesser degree, land extraction, agriculture, livestock, and hunting were identified at 64, 45, 14, and one point, respectively, which represent less than 5% of the total sampled points. Although domestic dogs were not registered, several individuals in the area could potentially pose a threat to the leporid species. Figure 3 illustrates the canonical correlation analysis (CCA) plot, depicting the relationship between the volcano rabbit, eastern cottontail, and Mexican cottontail, and environmental pressure factors.

The eigenvalues of the first and second species axes are 0.073 and 0.0312, respectively, indicating that the three species are segregated along these axes. Axis 1 accounts for 70.24% of the variance, while Axis 2 accounts for 29.76%. The red arrows indicate the total variance explained by each variable, thus acting as a good proxy of their importance. The first component (Axis 1) exhibited positive correlations with the following variables: the number of mature trees (M), houses (Ho), roads (Ro), agriculture (Ag), fires (F), and material extraction (Ex). The second component (Axis 2) exhibited negative correlations with houses (Ho), extraction of material (Ex), and open-air dumps (O) as habitat variables. The presence of rocks (Rk), young trees (Y), deforestation (D), and agriculture (Ag) were the determining factors explaining the most variation in the presence of the three leporid species, as indicated by the larger arrows in the diagram. It is noteworthy that human activities, like deforestation, fires, agriculture, and firewall breaches, impacted the presence of the three species. The interested reader can find maps of reforestation, agriculture, trash, and deforestation in the supplementary material; see Figures A2 to A5. In general, agricultural activities were limited to lower heights and consisted mainly of potato and oat fields. Most fecal pellets were found in areas with high densities of bunch grassland, rocky areas, and a certain amount of exposed soil. The presence of the Mexican cottontail and eastern cottontail species is favored by the abundance of young trees, but the volcano rabbit presence is negatively correlated with young trees. Also, the presence of shrubs is important for the volcano rabbit, but it does not seem to be a determining factor for eastern cottontail and Mexican cottontail.

## 3.3 Variation of precipitation and vegetation

**Fig. 4:**
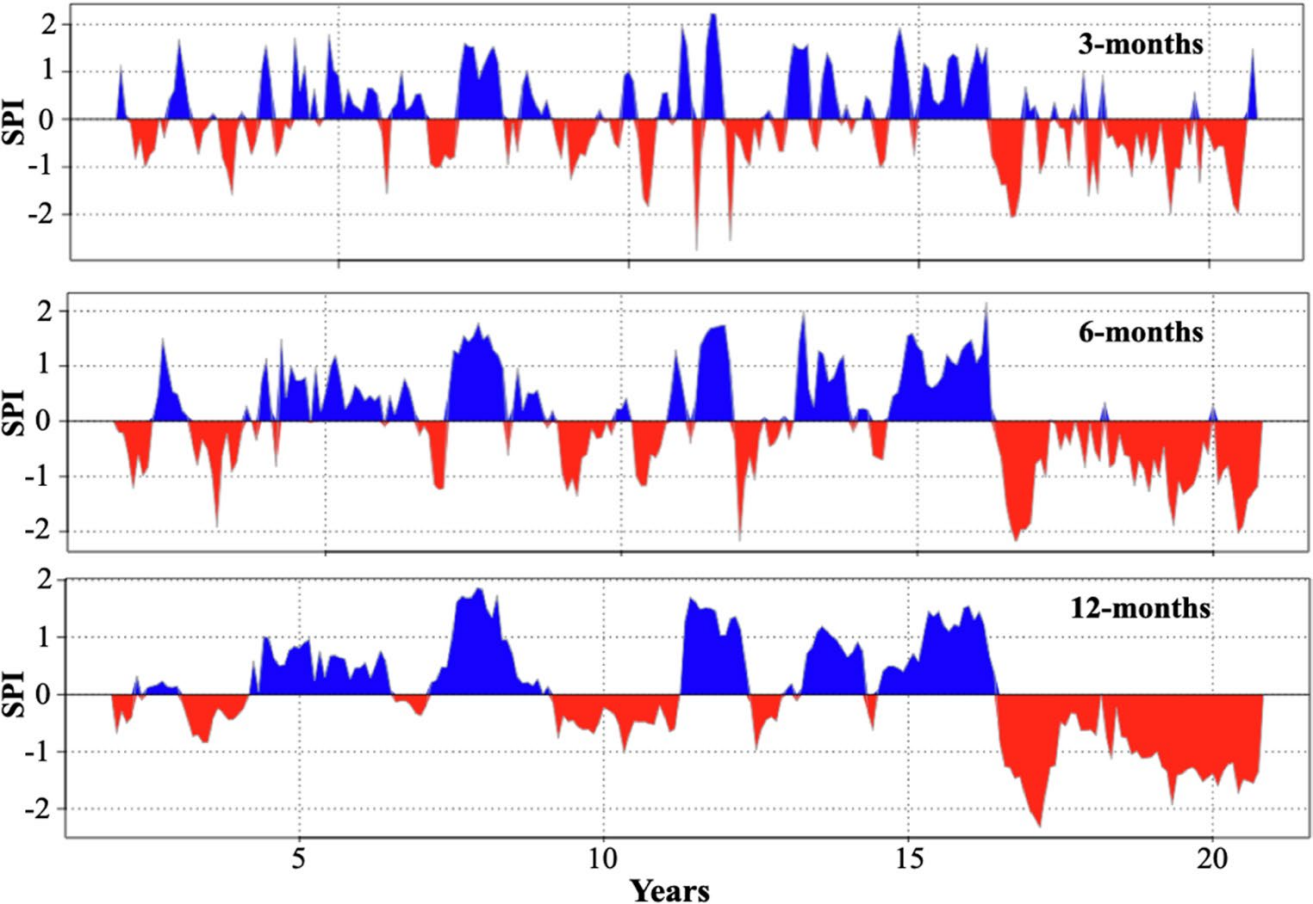
Standardized Precipitation Index (SPI) in the last 20 years (2000–2020) in the habitat of the volcano rabbit, eastern cottontail, and Mexican cottontail. Three accumulation periods, by 3, 6, and 12 months, are depicted. Positive values of SPI (blue-shaded areas) represent high humidity conditions, whereas negative SPI values (red-shaded areas) indicate drought conditions

The SPI showed unitless values ranging from − 2 to 2. The positive values of the SPI represent high humidity conditions, while negative values are related to drought conditions. Figure 4 shows the Standardized Precipitation Index (SPI) for the 2000–2020 period over the study area, with accumulation periods of 3, 6, and 12 months. Accordingly, it is observed that over the last four years, the volcano rabbit’s habitat has been characterized as a drought zone, indicating that precipitation levels in 2018 were lower than the mean for the period 2000–2020.

**Fig. 5:**
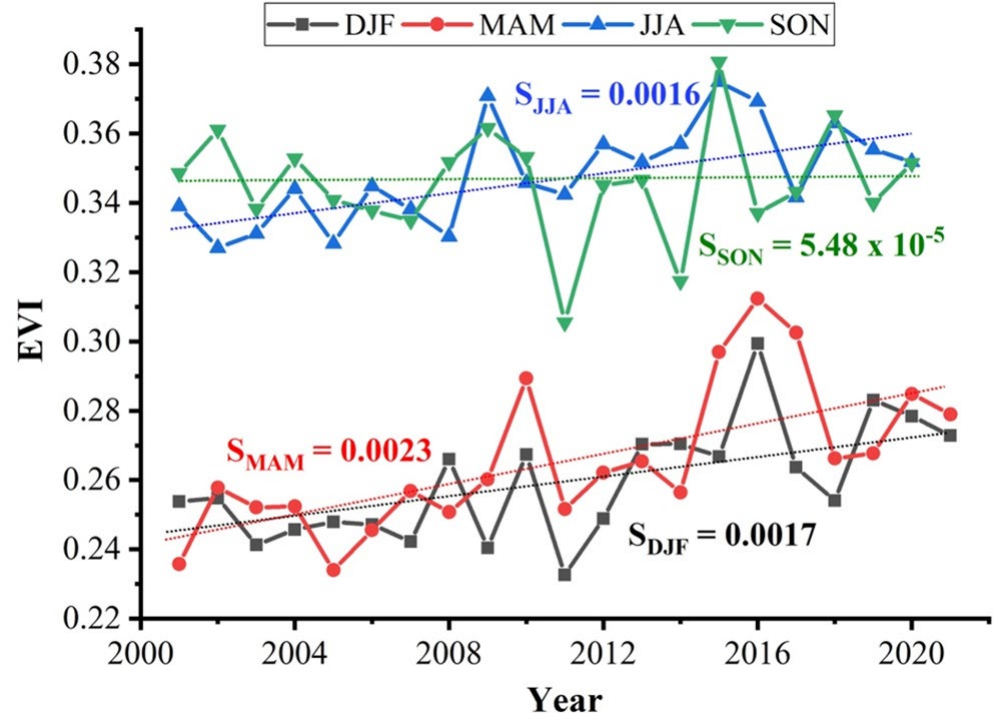
Seasonal distribution of the (unitless) enhanced vegetation index improved (EVI) during 2000–2020. The S_*i*_ parameter, on which *i* means any of the quarters: DJF (December to February), MAM (March to May), JJA (June to August), or SON (September to November), represents the slope of the corresponding quarterly trend line

Regarding vegetation development patterns over the study area during the 2000–2020 period, the EVI reveals a separation of vegetative development into two distinct groups. The first group has elevated EVI values, as seen during the rainy seasons (JJA and SON). As expected, the lowest values were observed in the dry-cold (DJF) and dry-hot (MAM) seasons. Figure 5 shows the quarterly mean distributions (DJF, MAM, JJA, SON) of the EVI over the study area for the 2000–2020 period. The patterns in vegetation development during the rainy season have remained relatively constant over the past 20 years, as the trend slope is near zero (S_SON_ = 5.84 × 10^− 5^ yr^− 1^). Besides, the SAI during the rainy season of 2018 has low values, ranging between 0.14 and 0.20; this means that vegetation remained the same in the rainy season (2018) compared with the whole period (2000–2020).

During the dry-hot season, the EVI trend increases slightly but continuously (S_MAM_ = 2.23 × 10^− 3^ yr^− 1^) and shows a sudden maximum in 2016. Despite the droughts, a reforestation campaign referred to by local communities may cause the EVI to reach a maximum in 2016. It is worth noting that EVI or NDVI products did not differentiate between vegetation types and features, such as young or old trees.

## 4. Discussion

The integration of citizen science, ecology, and habitat change in the leporids of the central mountains of Mexico enabled us to observe a trend of the volcano rabbit shifting to higher elevations over time. It makes sense to consider that in the region, the lower elevations are close to towns, roads, highways, and reforestation activities, features that limit the presence of this endangered species (Uriostegui-Velarde et al. 2018). In contrast, areas with a higher percentage of grassland are located at higher elevations (Osuna et al. 2022). These factors are particularly pertinent in the Sierra Chichinautzin and adjacent regions (Uriostegui-Velarde et al. 2018). Resources such as food, breeding sites, and shelter play a vital role in defining the spatial distribution, habitat utilization, and home range of species (Velazquez and Gopar-Merino 2018). In this sense, the volcano rabbit is a habitat specialist, relying on high-elevation areas with dense bunchgrass and shrub layers to evade predators (Rizo-Aguilar et al. 2015), where communities of *Festuca tolucensis* and *Trisetum altijugum-Festuca tolucensis* dominated (Velazquez and Heil 1996). In contrast, although the Mexican cottontail has a declining population trend, it prefers reforested areas, young trees, and areas with limited deforestation (Lorenzo and Lanier 2019). Similarly, the eastern cottontail was often found in disturbed areas, including agricultural zones, material extraction sites, roads, and houses (Nielsen and Lanier 2019). For the above, cottontail species have been demonstrated to be habitat generalists, in agreement with what has been reported for this species (Osuna et al. 2022). This article sheds light on the environmental factors that influence the local distribution of three leporid species, highlighting the differences in these factors for each species, in particular address that the presence of the volcano rabbit is positively correlated with the abundance of shrubs and the absence of young trees. This feature makes it more vulnerable, compared to the other two species, to reforestation campaigns that occurred some years before the study. On this regard, the reforestation is a new factor that influence the distribution of volcano rabbit not considered before (Uriostegui-Velarde et al. 2018). Likewise, individual volcano rabbits exist in isolated patches throughout the region. According toVelazquez (1994) the densities for the volcano rabbit were 0.11 to 1.20 rabbits ha^− 1^, while the abundance is from 0.1 to 3.1 latrines m^− 2^ (Velazquez and Heil 1996). However, these proportions could be lower in 2018 due to the factors mentioned above, with populations distributed in isolated patches across the landscape.

The public policies promoting reforestation and barriers to population dispersal (like Highway 95D, Mexico City to Cuernavaca) exacerbate the volcano rabbit’s vulnerability (Uriostegui-Velarde et al. 2015), which finds food and protection in bunchgrass habitat (Cervantes and Martinez 1992; Hunter and Cresswell 2015). In public perception, reforestation is an activity that benefits nature. However, in the case of some species of leporids, this perception is incorrect. We observed no individuals present, or traces of excrement that over time began to degrade, in sampling points with more than 15 trees within a 10 m radius, corroborating previous findings that closed forests reduce volcano rabbit abundance (Hunter and Cresswell 2015; Rizo-Aguilar et al. 2015). Unfortunately, several areas of alpine grassland suitable for the volcano rabbit have been transformed into highly dense forested areas with a slight separation between them. It is also possible to predict that colonies within high tree-density areas will disappear due to grassland drying out, like other species worldwide (Carro et al. 2019).

Likewise, reforestation has been reflected in an increase in EVI during hot, dry seasons; however, during the rainy season, EVI has remained relatively constant over the last 20 years. Lastly, a possible reason is that reforestation is combined with a decrease in precipitation over the previous 4 years (see Fig. 5); if precipitation had remained unchanged in 2018, we would also expect an increase in EVI during the rainy season (SON). However, the decrease in rainfall over the last 4 years (see Fig. 5) is compensated for by reforestation. As a result, the EVI (rainy season) remains unchanged. From the comments of the area’s inhabitants, we learned that in 2015, an intense reforestation campaign took place. Young trees began to mature and generate more shade, causing the small shrubs to decline, which is important since, as we saw, the species prefers young trees over an abundance of mature trees.

Citizen science is a tool that has grown in political discourses and on digital platforms, and this has benefits and challenges in each research (Dickinson et al. 2012). Factors such as formal education, gender, age, religion, existing knowledge, and interests of the target audience can directly influence motivation in participants and attitudes toward the project (Singh and Rahman 2012; Crall et al. 2013). During the training sessions for this project, participants were educated on various aspects, including the significance of the area and its species, monitoring techniques, species differentiation, conservation efforts for leporid species, and the use of camera traps and cameras. As a challenge, we identified that the high turnover of brigade participants posed an obstacle to long-term community involvement and knowledge transfer. An active training approach is recommended to help cultivate interest among participants, leading to more enduring effects and behavioral change (Williams et al. 2012). As a benefit, the participant played a vital role in identifying issues in the area and assessing landscape management policies implemented by local authorities. For instance, the community received more financial compensation (USD 100 ha^− 1^) for leasing their land annually than ecosystem services payments. However, land leasing posed a significant challenge to the conservation of the volcano rabbit, Mexican cottontail, and eastern cottontail species, as land lessees often employed aggressive agrochemical packages for potato cultivation.

Recognizing the citizen challenge, ecology of the leporids, and landscape habitat change, several actions are essential for developing effective conservation strategies. These strategies may include: (1) Citizen Science: Engaging local communities in monitoring programs to increase awareness of conservation issues and promote scientific literacy. This approach also fosters community engagement and strengthens governance in the area. (2) Species Distribution Mapping: Conducting comprehensive surveys to determine population distribution and implement conservation measures focused on enhancing habitat connectivity and minimizing human disturbances. (3) Continuous Monitoring: Sustaining efforts to monitor all three species to assess their responses to habitat changes and investigate potential displacements resulting from interactions with other species. (4) Habitat Change and Drought Monitoring: Evaluating the impact of human disturbances and climate change on habitat alteration, particularly in grassland areas critical for the volcano rabbit’s survival. (5) Collaboration with regional governments and local authorities is essential for effectively researching and protecting endangered species. This collaboration involves forming monitoring groups, providing training, collecting data, and ensuring accurate reporting of findings. It would be desirable for them to obtain economic resources for conservation.

## 5. Conclusions

During fieldwork in the study area and using citizen science tools, it was possible to register the presence of the volcano rabbit, eastern cottontail, and Mexican cottontail. The habitat conditions observed for the three species were generally consistent with their natural distribution. However, the data suggest that the volcano rabbit was more sensitive to anthropogenic activities and climate change than the other two species. In this sense, several hypotheses linked with land-use change, reforestation activities, and a decrease in precipitation may have affected the local distribution of the volcano rabbit. Nevertheless, this did not allow us to conclude definitively that the Mexican cottontail and eastern cottontail have better adaptive qualities on the Pelado Volcano than the volcano rabbit. Likely, a greater number of sites, sampling through multiple years, and other methods of monitoring can be added during additional research.

Finally, several strategies aimed at promoting the conservation of the volcano rabbit were proposed, including: (i) engaging local communities in monitoring programs, (ii) determining the distribution of the species, (iii) assessing its responses to habitat changes, and (iv) favoring the collaboration between regional governments and local authorities to create effective management plans.

## Supplementary Information

Below is the link to the electronic supplementary material.

Supplementary Material 1

## Data Availability

No datasets were generated or analysed during the current study.
