# Supplementary material for: Connecting the dots: integrating citizen science, ecology, and habitat change for the conservation of three Leporidae species in the central mountains of Mexico
Source: Naturwissenschaften. 2025 Dec 1;112(6):95. doi: 10.1007/s00114-025-02048-1 (PMC12669319; doi:10.1007/s00114-025-02048-1)
Supplement: Supplementary file 1 — Supplementary Material 1 [file 114_2025_2048_MOESM1_ESM.pdf]

Appendix

Figure legends (Appendix)

Fig.A1. Sort of soil type and vegetation in the study area.

Fig.A2. Evidence of deforestation near the transects in the study area.

Fig.A3. Evidence of reforestation near the transects in the study area.

Fig.A4. Evidence of agriculture near the transects in the study area.

Fig.A5. Evidence of trash near the transects in the study area.

Figures (Appendix)

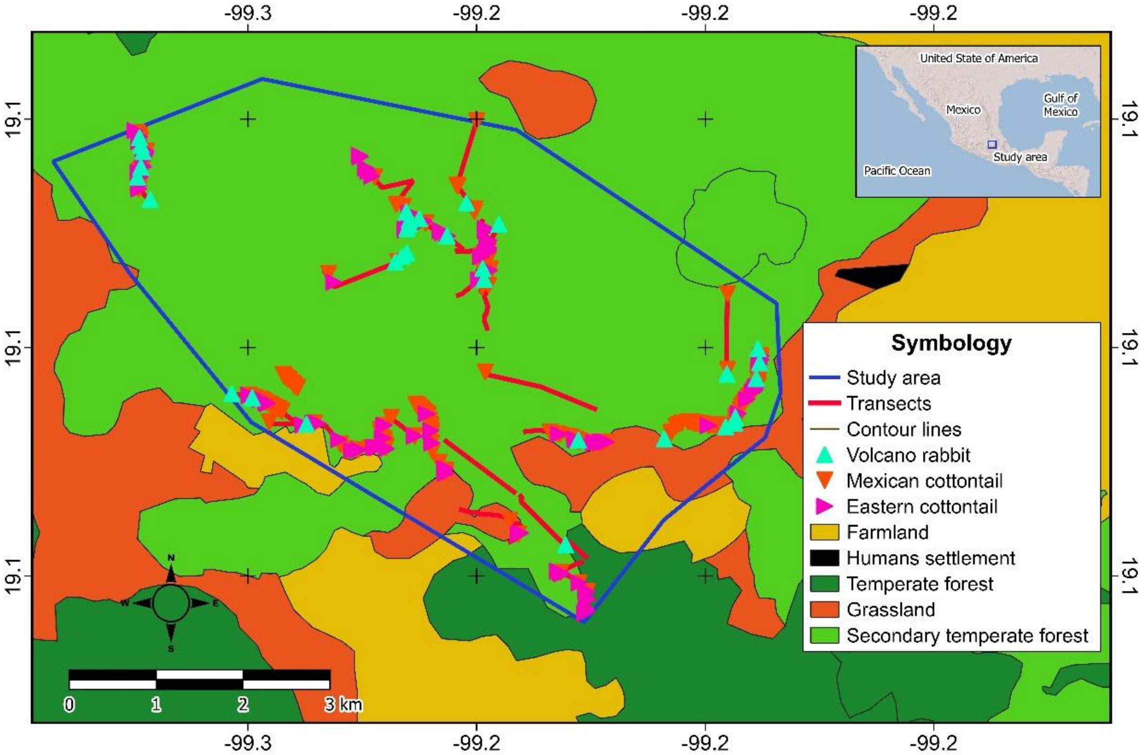

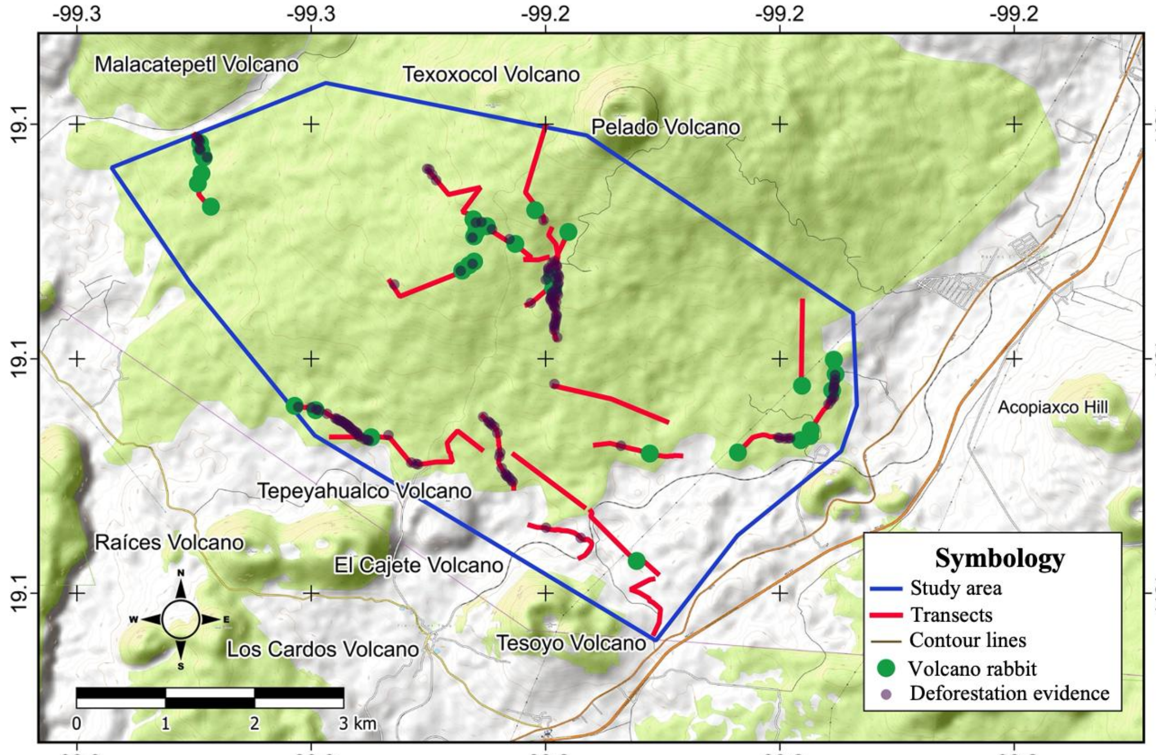
-99.3
-99.3
-99.2
-99.2
-99.2
19.1
19.1
19.1
Malacatepetl Volcano
Texoxocol Volcano
Pelado Volcano
Acopiaxco Hill
Tepeyahualco Volcano
Raíces Volcano
El Cajete Volcano
Los Cardos Volcano
Tesoyo Volcano
N
S
E
W
0
1
2
3 km
Symbology
Study area
Transects
Contour lines
Volcano rabbit
Deforestation evidence

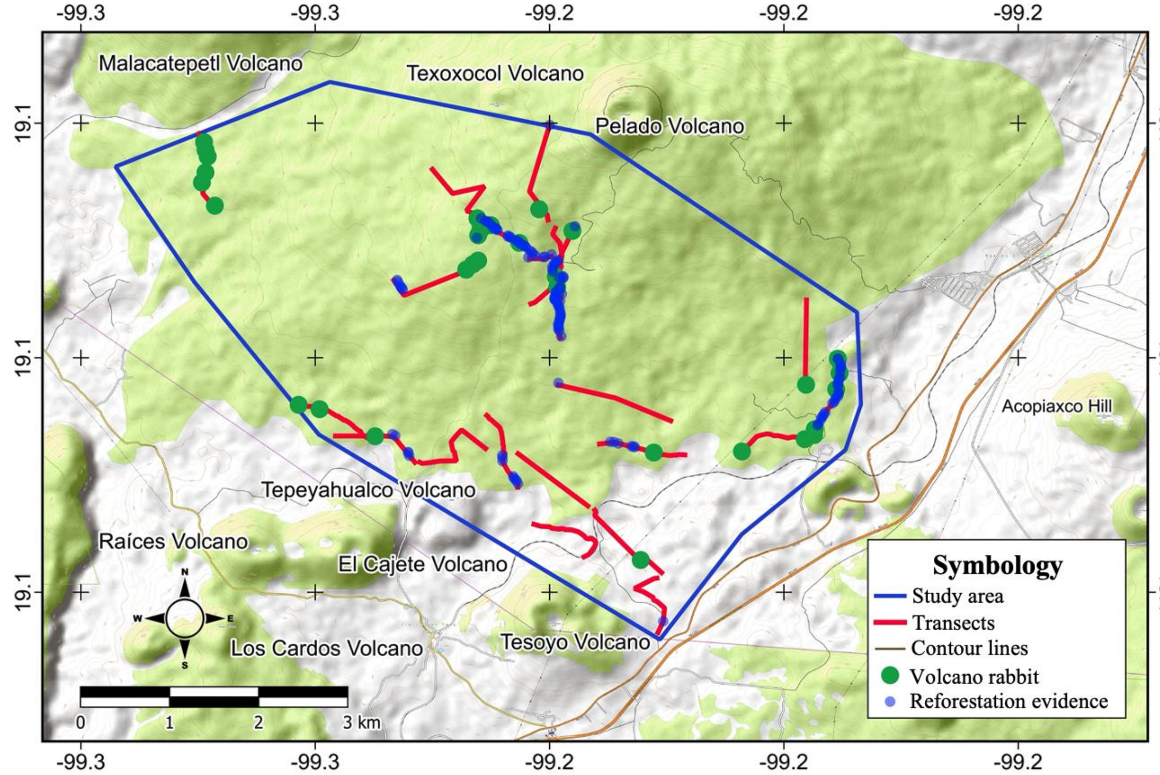
-99.3
-99.3
-99.2
-99.2
-99.2
19.1
19.1
19.1
Malacatepetl Volcano
Texoxocol Volcano
Pelado Volcano
Acopiaxco Hill
Tepeyahualco Volcano
Raíces Volcano
El Cajete Volcano
Los Cardos Volcano
Tesoyo Volcano
N
S
W
E
0
1
2
3 km
Symbology
Study area
Transects
Contour lines
Volcano rabbit
Reforestation evidence

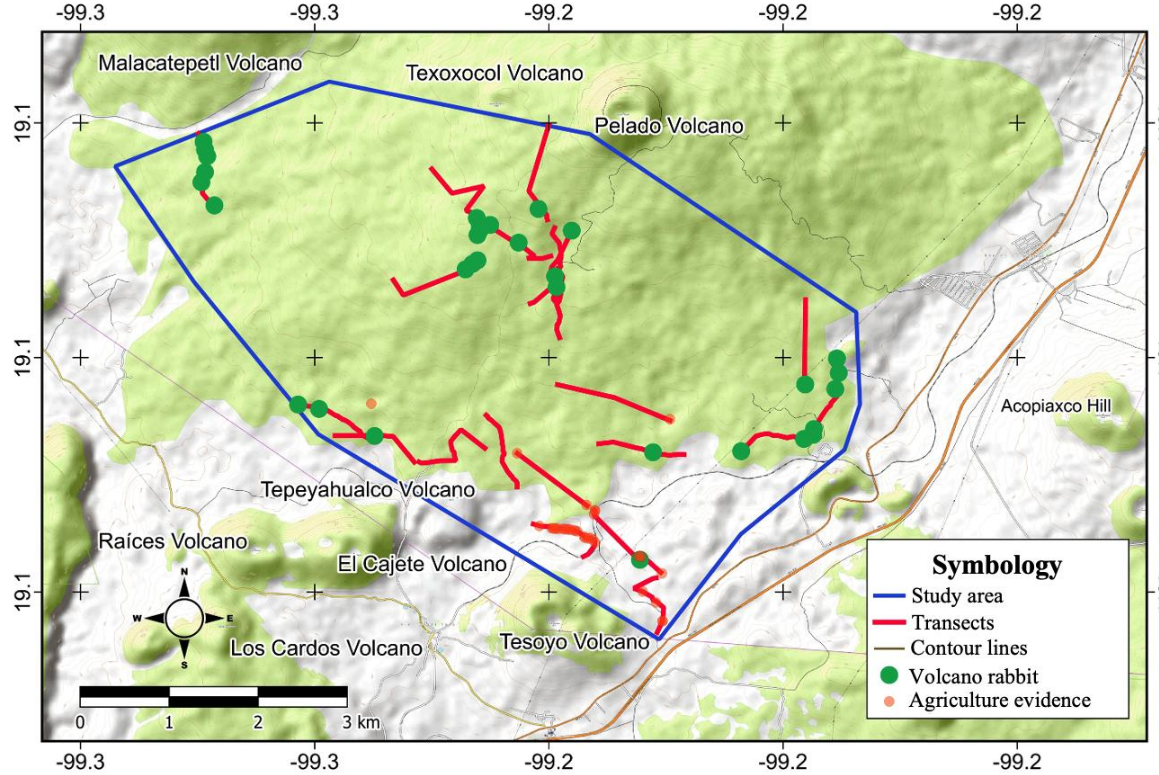
-99.3
-99.3
-99.2
-99.2
-99.2
19.1
19.1
19.1
Malacatepetl Volcano
Texoxocol Volcano
Pelado Volcano
Acopiaxco Hill
Tepeyahualco Volcano
Raíces Volcano
El Cajete Volcano
Los Cardos Volcano
Tesoyo Volcano
N
W
E
S
0
1
2
3 km
Symbology
Study area
Transects
Contour lines
Volcano rabbit
Agriculture evidence

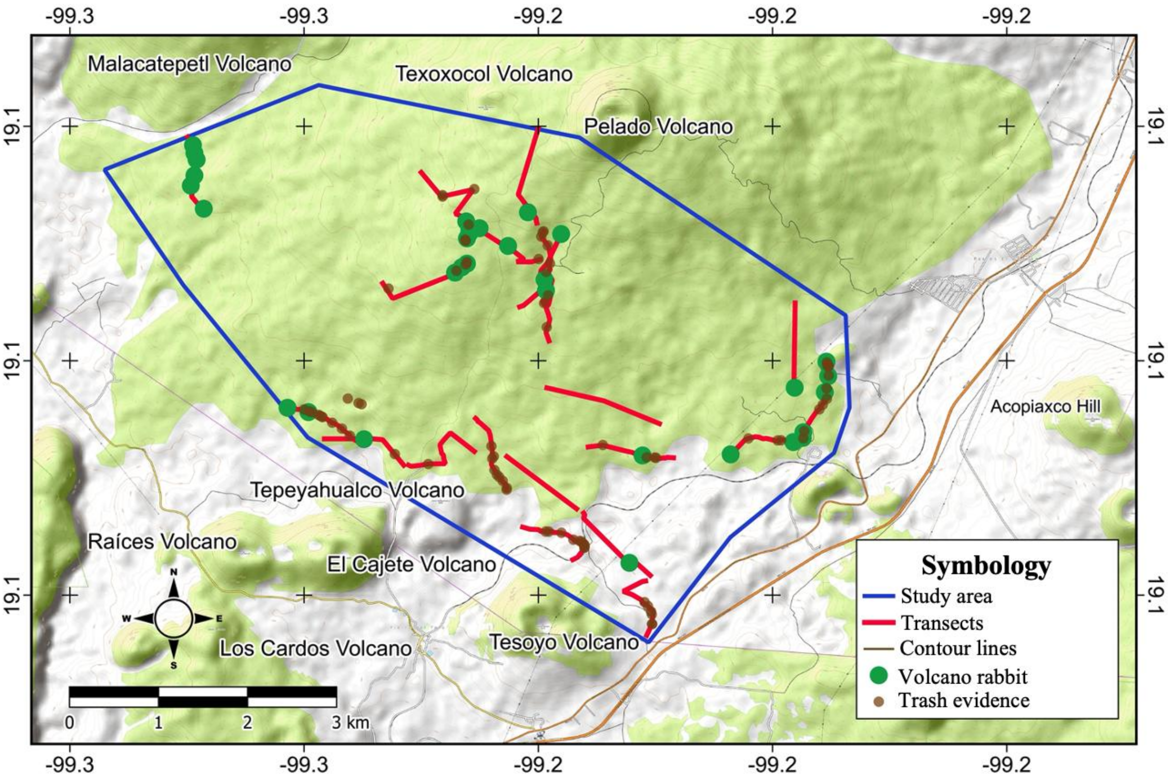
-99.3
-99.3
-99.2
-99.2
-99.2
19.1
19.1
19.1
Malacatepetl Volcano
Texoxocol Volcano
Pelado Volcano
Acopiaxco Hill
Tepeyahualco Volcano
Raíces Volcano
El Cajete Volcano
Los Cardos Volcano
Tesoyo Volcano
N
W
E
S
0
1
2
3 km
Symbology
Study area
Transects
Contour lines
Volcano rabbit
Trash evidence
